# Generic competition and price developments in the USA, Germany and Switzerland (2007–2023): a longitudinal observational study

**DOI:** 10.1136/bmjph-2024-002535

**Published:** 2025-09-17

**Authors:** Simon Hediger, Luca Locher, Kerstin Noëlle Vokinger

**Affiliations:** 1 Academic Chair for Regulation in Law, Medicine and Technology, Department of Health Sciences and Technology, ETH Zurich, Zurich, Switzerland; 2 Academic Chair for Regulation in Law, Medicine and Technology, Faculty of Law, University of Zurich, Zurich, Switzerland; 3 Epidemiology, Biostatistics and Prevention Institute, Faculty of Medicine, University of Zurich, Zürich, Switzerland

**Keywords:** Pharmacoepidemiology, economics, Health Services Accessibility

## Abstract

**Introduction:**

Drug expenditure has increased over the past years in the USA and Europe, mainly driven by originator drugs. Generic drugs have a substantial cost-saving potential. We analysed the number of generic competitors entering the market over time, distribution of market share between originator drugs and their generic competitors and association between the number of generic competitors and price changes of originator and generic drugs in the USA, Germany and Switzerland.

**Methods:**

In this longitudinal observational study, we included all originator drugs and their corresponding generic competitors with market entry between 2007 and 2023 in the USA, Germany and Switzerland from the IQVIA database. We extracted quarterly price and sales volume data for the same study period from the IQVIA database. Descriptive statistics and regression analyses were conducted to answer the research questions.

**Results:**

530 active substances in the USA, 406 in Germany and 108 in Switzerland were included. 3 years after competition start, an average of four generic competitors entered the market in the USA, 7.6 in Germany and 3.3 in Switzerland. An increase in the number of generic competitors was associated with an increase in average generic market share. On average, the generic market share reached 90% after the entry of five generic drugs in the USA and nine in Germany, and was not reached in Switzerland. An association between the number of competitors and relative price decrease of generic drugs was observed in all three countries. This association was also observed for originator drugs in Switzerland and Germany, but not in the USA.

**Conclusions:**

The findings indicate that policies targeting market entry and prices of generic drugs would be helpful in all three countries. More research is needed to assess the impact of previous and future policies.

**WHAT IS ALREADY KNOWN ON THIS TOPIC**

- Drug expenditure has increased over the past years in the USA and Europe, mainly driven by originator drugs.
- Generic drugs have a substantial cost-saving potential.

**WHAT THIS STUDY ADDS**

- Differences in generic competition and price developments were observed between the USA, Germany and Switzerland.
- The increase of generic competitors was fastest and the total number of competitors largest in Germany, while in the USA, the average generic market share showed the steepest increase as the number of competitors grows.
- An association between the number of competitors and relative price decrease of generic drugs was observed in all three countries.

**HOW THIS STUDY MIGHT AFFECT RESEARCH, PRACTICE OR POLICY**

- All three countries could focus on policy changes that would improve market entry and affordability of generic competitors.
- The findings indicate that policies targeting market entry and prices of generic drugs would be helpful in all three countries.
- More research is needed to assess the impact of previous and future policies.

## Introduction

 Drug expenditure has increased over the past years in the USA and European countries. This spending is mainly driven by brand name drugs, also referred to as originator drugs.^1^ Studies have demonstrated the cost-saving potential of generic drugs.^2^,^6^ They may enter the market after the originator drug’s patents and regulatory exclusivities have expired.^2^ Generic drugs are bioequivalent replicas of their corresponding originator drug.^3 4^ They contain the same active ingredient and have identical quality, safety and efficacy profiles. Differences are limited to inactive ingredients, for example, colouring, flavouring or stabilising agents.^3 4^

Because generic drugs can help affordability and improve access of patients to drugs, governments across countries have developed different strategies to incentivise market entry and uptake of generic drugs.^7^ For example, the US Department of Health and Human Services released in October 2024 a sample list of generic drugs that the agency intends to include under the proposed Medicare US$2 Drug List Model.^8^ This would enable patients enrolled in Medicare prescription drug coverage to access these generic drugs for a low, fixed co-payment of no more than US$2 for a month’s supply per drug that target common conditions, such as high cholesterol and high blood pressure.^8^ In Germany, policymakers discussed the mandatory storage of generic drugs to secure sufficient supply for patients.^9^ And in Switzerland, co-payment for originator drugs increased from 10% to 40% as of January 2024 if a generic alternative is available.^10^ The objective is to achieve a cost reduction of approximately US$275 million per year.^10^

Prior studies focused on generic competition in the USA or on generic competition across different countries for a specific year.^4^,^711 12^ An analysis of generic competition over a longer time period and across countries with different healthcare systems could help to identify possible solutions to improve access to generic drugs. The objective of this study was to analyse the number of generic competitors entering the market over time, the distribution of market share between originator drugs and their generic competitors and the association between the number of competitors and price changes of originator and generic drugs in the USA, Germany and Switzerland.

## Methods

### Data sources and extraction

We identified all originator drugs that were marketed between 1 January 2007 and 30 September 2023 in the USA, Germany and Switzerland using the IQVIA Pricing Insights and MIDAS databases. Germany and Switzerland were included as additional countries because they are economically similar to the USA but pricing policies for generic drugs vary across all three countries. In the USA and generally also in Germany, manufacturers are free to set the prices of generic drugs.^13^ In Switzerland, generic prices depend on the market volume of the corresponding originator drugs. Prices of generic drugs must be at least 20% lower than the corresponding originator drug if the yearly average market volume of the originator drug is CHF (Schweizer Franken, Swiss Francs) 4 million or lower and at least 70% lower if the yearly average market volume of the originator drug exceeds CHF 25 million.^14^

We identified all originator drugs that had at least one generic competitor entering the market during this time period. A generic competitor was considered as entering the market if the IQVIA MIDAS database provided sales data for that drug. A drug is defined by a combination of active ingredient, package size and dosage form (route of administration).^12^ A generic drug was qualified as a competitor if the following characteristics were identical with the originator drug: active substance, package size and dosage form.^12^

We included the originator drugs and corresponding generics that matched the inclusion criteria in our study cohort and collected the following additional information: therapeutic area (based on the WHO’s Anatomic Therapeutic classification system) and date of generic market entry.

We referred to an originator drug and its corresponding generic competitor(s) as a competition group. Competition groups without any generic drugs entering the market were excluded.

We extracted quarterly price and sales volume data for the same study period from the IQVIA Pricing Insights and MIDAS databases for the drugs in our study cohort for the USA, Germany and Switzerland.

### Statistical analysis

We applied descriptive statistics to analyse the number of generic competitors entering the market between 1 January 2007 and 30 September 2023 in the USA, Germany and Switzerland.

Within each competition group, we calculated the generic market share for each number of competitors. Generic market share was defined as the total units of the active ingredient sold for generics divided by the sum of the total units of the active ingredient sold for generics and the originator drug.

We analysed the association between the number of competitors and price developments as follows: the drugs in the study cohort were categorised into three groups. The first group included the prices for all quarters within the study period for the originator drugs. The second group comprised the prices for all quarters within the study period for the generic drugs. For each quarter, we calculated the generic price as a quantity-weighted average of all generic drugs within a competition group. The third group included an overall market price series, that is, we calculated for each quarter a quantity-weighted average price, including both the prices of the originator and the generics within a competition group. Next, to calculate relative price developments, we determined for each competition group the percentage price change for the originator drugs, the corresponding generic drugs and the overall market. We achieved this by subtracting the price of the originator in the last quarter prior to competition from the price of the respective price series for each following quarter and for each country. The resulting differences were divided by the originator’s price in the last quarter prior to competition. Additionally, we calculated the sales volume prior to competition for each competition group and country by multiplying the originator’s price in the last quarter prior to competition with the quantity sold in that same period. To provide a descriptive overview of the association between number of competitors and price changes, we created boxplots for each group (originators, generics, all drugs) and country. We then estimated the effect of the number of competitors on the percentage price change using a fixed effects regression. We controlled for the number of competitors, the starting quarter of competition, the quarters since the start of competition, the therapeutic area and the sales volume prior to competition.

We excluded competition groups from this analysis that had more than 15 competitors in the USA, more than 24 competitors in Germany and more than 7 competitors in Switzerland, respectively. The reason was a lack of sufficient observations in those competition groups.

The 95% CIs for the regression coefficients were constructed via clustered and heteroskedasticity-consistent SEs. The clusters were formed on the active substance level.

A more formal explanation of the statistical analysis is outlined in the online supplemental appendix.

This study followed the Strengthening the Reporting of Observational Studies in Epidemiology reporting guideline.

All statistical analyses were conducted using R, V.4.4.0 (R Project for Statistical Computing).

## Results

### Overview

Overall, our study cohort included 530 active substances in the USA, 406 active substances in Germany and 108 active substances in Switzerland. This corresponds to 1825 originator drugs and 10 967 generics in the USA, 1855 originator drugs and 16 293 generics in Germany and 366 originator drugs and 1490 generics in Switzerland when aggregating originator drugs and generic drugs based on their active substance, package size and dosage.

**Figure 1:**
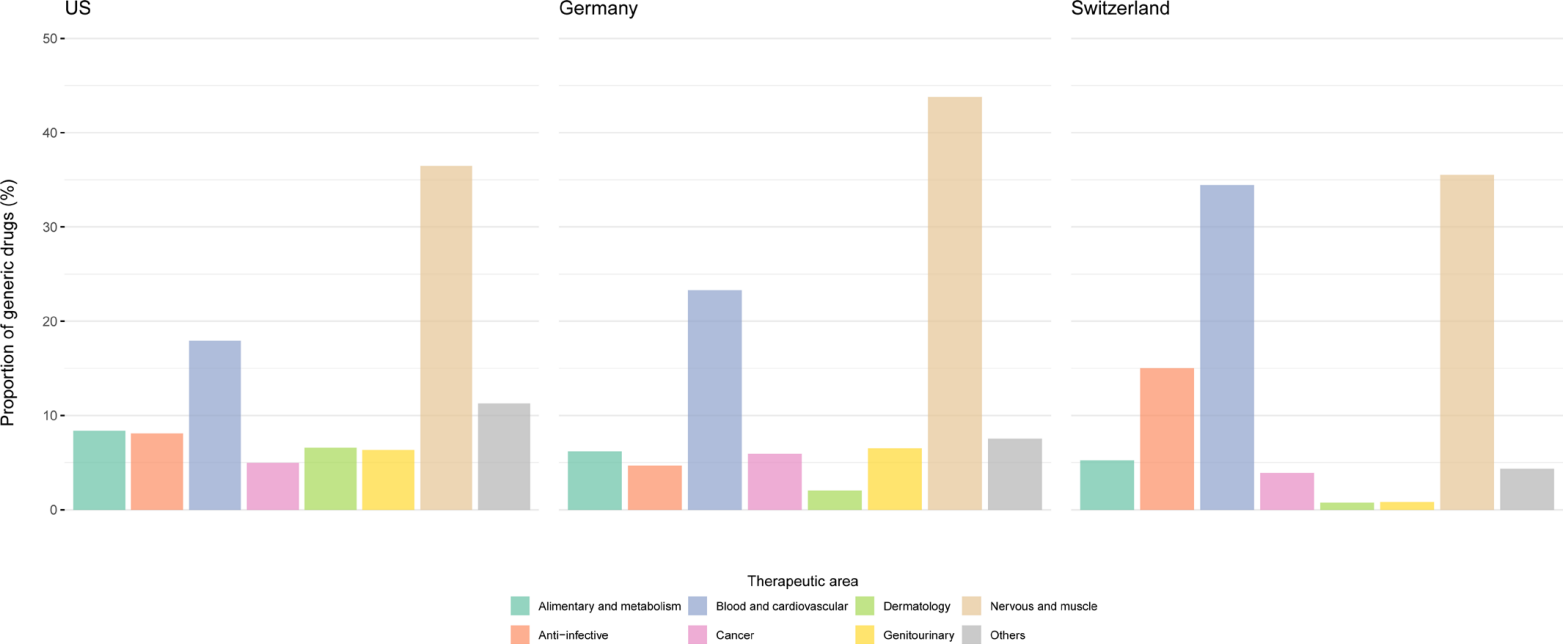
Overview of generic drugs and therapeutic areas. Notes: distribution of generic drugs across therapeutic area.

In all three countries, most of the generic drugs targeted diseases of the nervous system (3999 (36%) in the USA, 7134 (44%) in Germany and 529 (36%) in Switzerland), followed by drugs for treatment of blood and cardiovascular disorders (887 (18%) in the USA, 3793 (23%) in Germany and 513 (34%) in Switzerland) (figure 1).

### Generic competition

On average, the number of generic competitors for the corresponding originator drugs increased after the start of competition in all three countries. In the USA, 2.6 generic competitors were marketed on average the following quarter after start of competition, 3.3 generic competitors entered the market 1 year after start of competition, 4 generic competitors after 3 years and 4.5 generic competitors after 5 years. In Germany, 3.7 generic competitors entered the market on average the following quarter after start of competition, 6.3 generic competitors after 1 year, 7.6 generic competitors after 3 years and 8 generic competitors after 5 years. In Switzerland, 2.6 generic competitors were on average on the market the following quarter after start of competition, 3 generic competitors after 1 year, 3.3 generic competitors after 3 years and 3.5 generic competitors after 5 years (table 1).

**Table 1:** Overview of generic competition in USA, Germany and Switzerland

Country	one quarter	1 year	2 years	3 years	4 years	5 years
USA	2.58	3.31	3.78	4.03	4.30	4.45
Germany	3.72	6.25	7.08	7.60	7.93	7.99
Switzerland	2.62	3.01	3.21	3.30	3.41	3.54

Notes: the table depicts the mean number of generic competitors one quarter, 1 year, 2 years, 3 years, 4 years and 5 years after start of competition.

### Generic market share

In general, an increase in the number of generic competitors was associated with an increase of average generic market share. The USA and Germany showed a concave increase pattern, whereas Switzerland demonstrated a linearly increasing generic market share.

**Figure 2:**
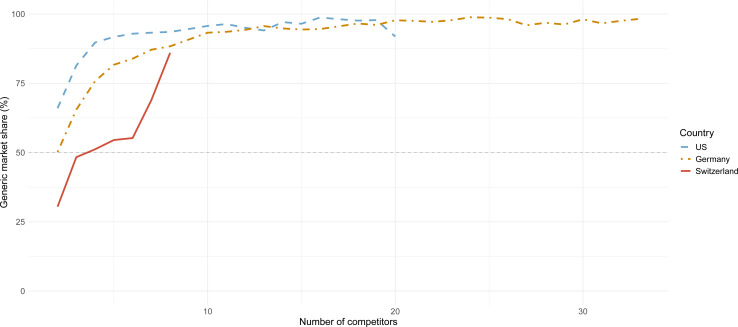
Average market share of generic drugs in the USA, Germany and Switzerland. Average market share of generic drugs (y-axis) based on the number of competitors (x-axis).

On average, generics accounted for 50% of the total market share after one generic competitor entered the market in the USA and Germany. For Switzerland, four generic competitors had to enter the market to reach an average market share of more than 50%. On average, the generic market share reached 90% after the entry of five generic drugs in the USA and nine in Germany. In Switzerland, the maximum average market share of 86% was reached after the entry of eight generic drugs (figure 2).

### Generic competition and price development

**Figure 3:**
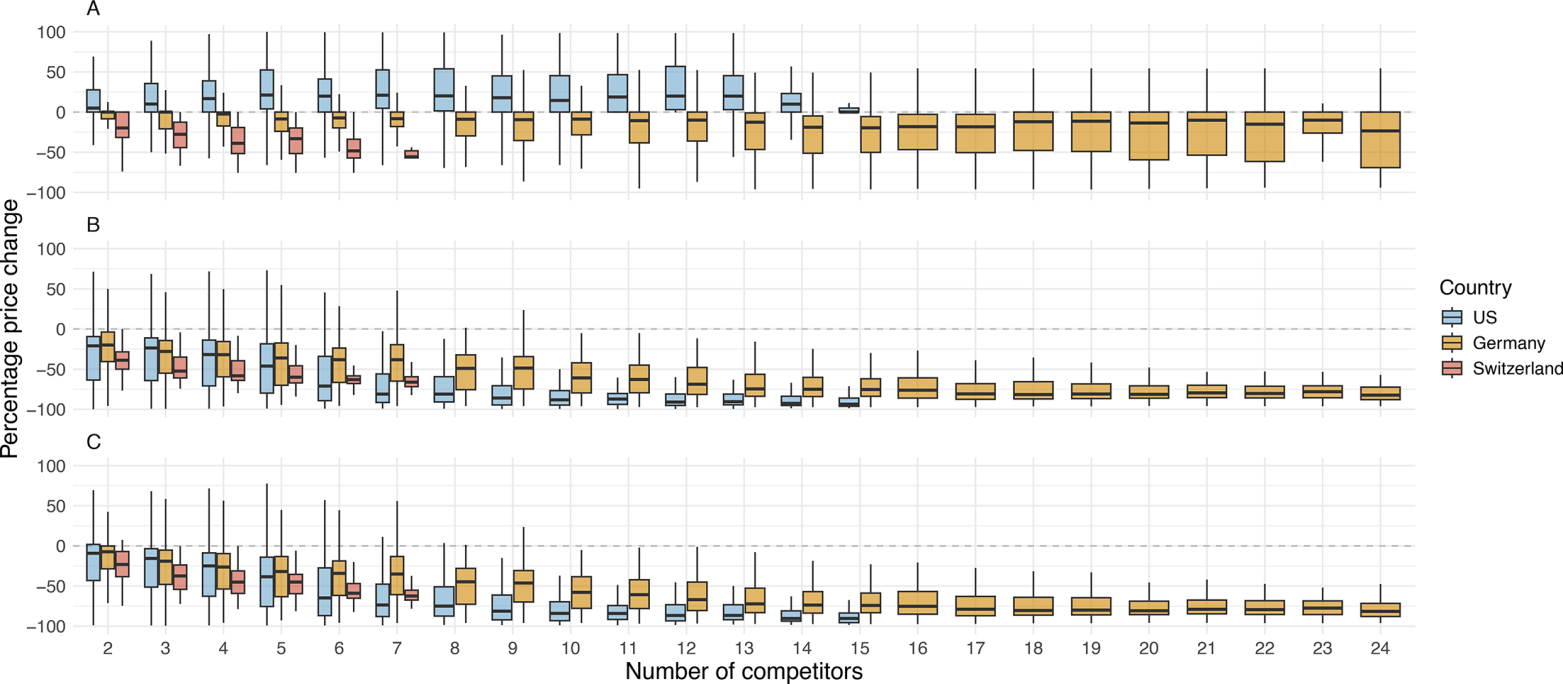
Generic competition and price development in the USA, Germany and Switzerland (descriptive analysis). Boxplots illustrate the percentage price change for each number of competitors for the originator drugs (panel A), generic drugs (panel B) and overall (originator and generic drugs; panel C).

An analysis of the median percentage price changes for originator drugs after the start of competition showed notable differences between the USA on the one hand and Germany and Switzerland on the other hand. In the USA, median percentage price changes increased slightly when more competitors entered the market. By contrast, median price changes for originator drugs were negative in Germany and Switzerland after market entry of the first generic competitor and prices continued to decrease with each additional generic competitor entering the market (figure 3A).

The first generic competitors entered the market with a median price difference of −20% in the USA, −19% in Germany and −39% in Switzerland compared with their corresponding originator drug. The median price changes for generic drugs decreased non-linearly with the increasing number of competitors entering the market (figure 3B).

The development of the median percentage price change of the overall group was similar to the price trends of the generic group (figure 3C).

**Figure 4:**
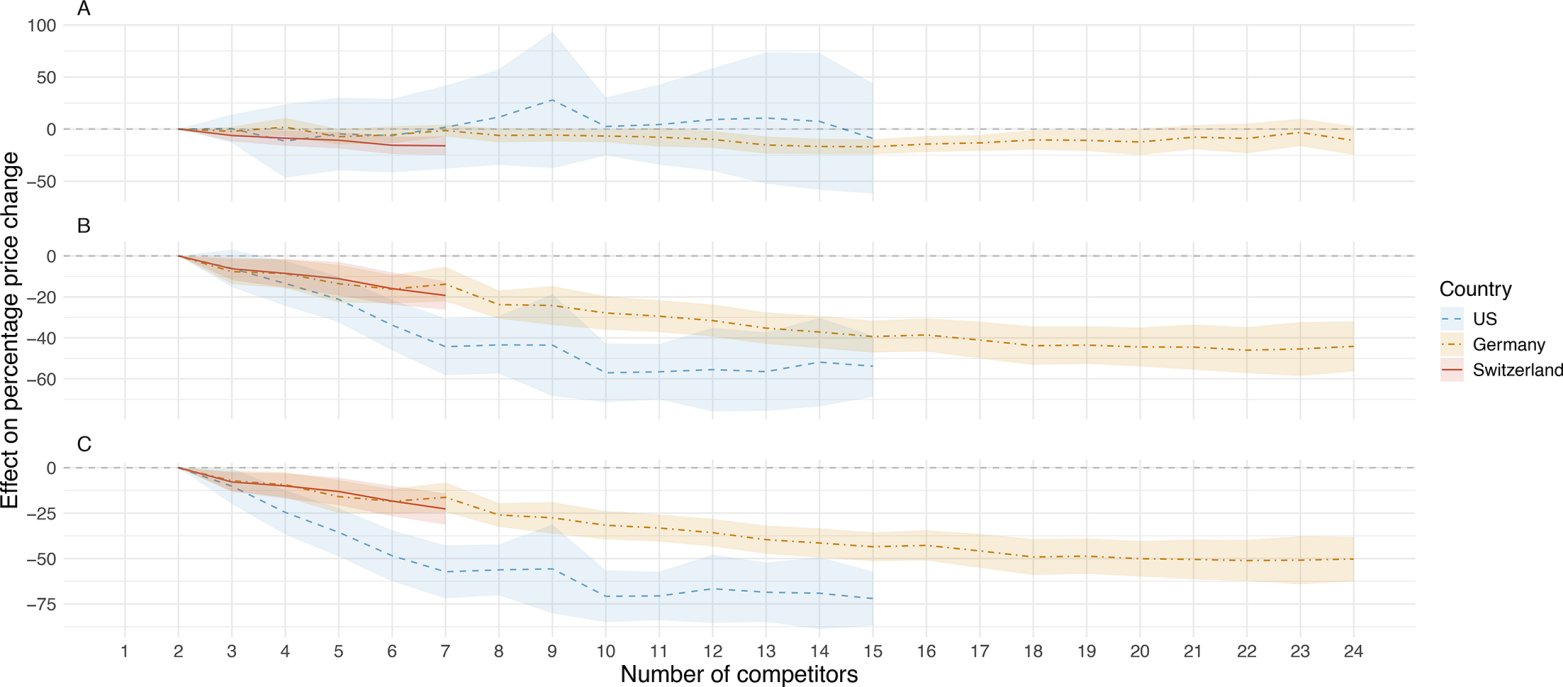
Effect of generic competition on price developments in the USA, Germany and Switzerland (regression analysis). Effect of generic competition on price developments (panel A: originator drugs, panel B: generic drugs, panel C: overall (originator drugs and generic drugs)) in the USA, Germany and Switzerland.

The results of the regression analysis showed that the estimated ceteris paribus effect of a second generic competitor on the percentage price change of originator drugs was on average 0.4 percentage points (%pt) (95% CI: (−13.2 to 14.0)%pt) in the USA, −2.2%pt (95% CI: (−6.5 to 2.2)%pt) in Germany and −6.2%pt (95% CI: (–11.8 to –0.5)%pt) in Switzerland (figure 4A).

When focusing on the generic group, the estimated ceteris paribus effect of a second generic competitor on the percentage price change was on average −6.1%pt (95% CI: (−15.2 to 3.0)%pt) in the USA, −7.6%pt (95% CI: (–13.7 to –1.6)%pt) in Germany and −6.3%pt (95% CI: (–11.8 to –0.7)%pt) in Switzerland (figure 4B). The estimated competition fixed effects of our regression analysis showed that the percentage price change in the generic pool decreased ceteris paribus on average with each additional generic competitor and stabilised after market entry of approximately 10 competitors in the USA. In Germany, a stabilisation of the competition fixed effects was observed after the entrance of approximately 20 generic competitors at a level of −44.4%pt (95% CI: (–53.9 to –34.9)%pt). In Switzerland, the estimated competition fixed effects decreased linearly, and reached −19.3%pt (95% CI: (–26.2 to –12.4)%pt) when six generic competitors entered the market (figure 4B).

The results of the regression analysis for the overall group showed similar trends to the findings for the generic group (figure 4C).

## Discussion

Differences in generic competition and price developments were observed between the USA, Germany and Switzerland. The increase of generic competitors was fastest and the total number of competitors largest in Germany, followed by the USA and Switzerland. The average generic market share showed the steepest increase as the number of competitors grows in the USA, followed by Germany and Switzerland. An association between the number of competitors and relative price decrease of generic drugs was observed in all three countries. This association was also observed for originator drugs in Switzerland (most pronounced) and Germany, but not in the USA.

Former studies showed that the number of generic competitors was positively associated with the market size.^15 16^ Our findings indicate that Switzerland and the USA had a comparable number of marketed generic competitors even though Switzerland is a much smaller market. By contrast, approximately double the number of generic competitors were marketed in Germany 1 year and later after market entry of the first generic competitor. Germany is a larger market compared with Switzerland but a smaller market compared with the USA. A possible explanation for the lower number of generic competitors in the USA could be the strategies of originator drug manufacturers that delay market entry of generic drugs, especially in the USA.^2^ Such strategies include, for example, secondary patenting, that is, obtaining new patents on peripheral aspects of the drug or its use. Reverse payment settlements, also referred to as pay-for-delay settlements, are another example of the delay of generic drugs by settling litigation. The most controversial kind of reverse payment settlement involves agreements in which generic manufacturers agree to drop their legal challenges to originator manufacturers’ patients and market their products later than might have been anticipated if their litigation was successful in return for compensation.^2 17 18^

Our findings demonstrate that the average generic market share continuously increased with every additional generic competitor that entered the market in the USA, Germany and Switzerland. However, differences were observed across countries. While, on average, 50% of the generic market share was reached after one generic competitor entered the market in the USA and Germany, four generic competitors were necessary in Switzerland to reach the same average market share. The generic market share reached, on average, 90% after five and nine generic drugs entered the market in the USA and Germany, respectively; however, an average market share of 90% was not reached in Switzerland. These diverging results could be driven by the different policies in these countries. In Germany, pharmacists are obliged to prioritise generic drugs, that is, exchange an originator drug prescribed by a physician for a cheaper generic drug, unless the physician explicitly excludes such an exchange. If the patient decides to choose the more expensive (originator) drug, the patient has to pay the additional costs that exceed the price of the cheaper drug out of pocket.^19^,^21^ Such a strict policy does not apply in Switzerland. However, since January 2024, co-payment for originator drugs increased from 10% to 40% if a generic drug is available.^10^ The objective is to incentivise the uptake of generic drugs and achieve a cost reduction for drug expenditure of approximately US$275 million per year.^10^ A study outlined the variation in state regulation of generic drug substitutions in the USA.^22^ The authors concluded that there is a need for optimising state drug product selection laws to promote generic substitution, which may promote generic uptake and reduce drug spending.^22^

A commonality across all three countries was the association between generic competition and relative price decrease of generic drugs, which was most pronounced in the USA. This price decrease trend also held for originator drugs in Switzerland (most pronounced) and Germany, but not in the USA. A possible explanation for the differences in price developments across the three countries could be the underlying pricing policies. The prices of generic drugs in Switzerland are regulated.^13^ More specifically, generic prices in Switzerland depend on the market volume of the corresponding originator drugs. Prices of generic drugs must be at least 20% lower than the corresponding originator drug if the yearly average market volume of the originator drug is CHF 4 million or lower and at least 70% lower if the yearly average market volume of the originator drug exceeds CHF 25 million.^14^ The different pricing policies could also explain the price trends of originator drugs in the USA, Germany and Switzerland. Prices of originator drugs are negotiated in Germany (6 months after market entry) and Switzerland.^23 24^ While very few drugs will be subject to price negotiation in the USA on a yearly basis as of 2026,^25^ manufacturers were free to set the prices of all originator drugs included in our study cohort.^1^ A study demonstrated that post-approval prices of non-cancer drugs were generally stable but continued to increase for cancer drugs after market entry in the USA.^26^

### Limitations

Sales and price data were extracted from IQVIA, which mainly focuses on list prices and does not account for confidential rebates. Thus, prices of drugs with confidential rebates were overestimated. We were not able to rely on SSR Health data, a database that estimates rebates in the USA, because SSR Health data is limited to originator drugs and listed companies. Furthermore, IQVIA’s sales data may be under-represented.^27^ However, IQVIA’s Pricing Insights and MIDAS databases are scientifically acknowledged, comprehensive and broadly used databases for studies focusing on drug prices and sales within and across countries.^2228^,^30^ Our sales and pricing data were not at indication level. As a result, if originator drugs retained patent protection for certain indications (ie, skinny labelling), the generic market share may be underestimated. Given the observational nature of our setting, there are likely numerous unobserved variables that simultaneously influence both the number of competitors and pricing dynamics over time within each competition group. As a result, we did not interpret the coefficients from our linear regressions as estimates of causal effects. Instead, they should be understood as associations. For example, changes in disease prevalence could increase demand for certain drugs, which in turn may attract more competitors and simultaneously affect pricing—thereby confounding the observed relationship between competition and prices.

## Conclusions

Differences in generic competition and price developments were observed between the USA, Germany and Switzerland. The increase of generic competitors was fastest and the total number of competitors largest in Germany, while in the USA, the average generic market share showed the steepest increase as the number of competitors grows. An association between the number of competitors and relative price decrease of generic drugs was observed in all three countries. This association was also observed for originator drugs in Switzerland and Germany, but not in the USA.

All three countries could focus on policy changes that would improve market entry and affordability of generic competitors. The comparative analysis indicates that price negotiation policies could be helpful, especially for originator drugs in the USA and generic drugs in Germany, while Switzerland could focus on policies that incentivise the uptake of generic competitors. Research that analyses the impact of current and future policies on generic competition is needed to understand which policies are best to incentivise market entry and pricing of generics.

## Supplementary material

10.1136/bmjph-2024-002535 online supplemental file 1
