# Supplementary material for: Generic competition and price developments in the USA, Germany and Switzerland (2007–2023): a longitudinal observational study
Source: BMJ Public Health. 2025 Sep 17;3(2):e002535. doi: 10.1136/bmjph-2024-002535 (PMC12458845; doi:10.1136/bmjph-2024-002535)
Supplement: online supplemental file 1 [file bmjph-3-2-s001.pdf]

Appendix

Formal explanation of methodological approach

For quarter $t \in \mathcal{C}_{j,k}$, within a country j and a competition group k, apart from the observed originator price sequence p_t^{O}, we created two additional price series. First, the generic price sequence p_t^{G} was calculated as follows: for each quarter we took the quantity weighted average of all generic prices via

$$p_t^{\mathrm{G}} = \frac{\sum_{f \in F_t} Q_f^{\mathrm{G}} \tilde{p}_f^{\mathrm{G}}}{\sum_{f \in F_t} Q_f^{\mathrm{G}}},$$

where F_t is the set of all generic firms at t, $\tilde{p}_f^{\mathrm{G}}$ is the local price of f and Q_f^{G} is the uptake of f.

Second, the overall market price p_t^{M} was calculated in each quarter as the quantity weighted average of all prices via

$$p_t^{\mathrm{M}} = \frac{\sum_{f \in F_t} Q_f^{\mathrm{G}} \tilde{p}_f^{\mathrm{G}} + Q_t^{\mathrm{O}} p_t^{\mathrm{O}}}{\sum_{f \in F_t} Q_f^{\mathrm{G}} + Q_t^{\mathrm{O}}}.$$

The percentage price change for the originator, the generic pool and the overall market was then calculated as

$$r_t^{\mathrm{l}} = 100 \frac{p_t^{\mathrm{l}} - p_{t^*}^{\mathrm{O}}}{p_{t^*}^{\mathrm{O}}},$$

where t^* marks the last quarter before the competition starts within a country and a competition group and $l \in \{\mathrm{O,G,M}\}$. Further, within each country and competition group we calculated the sales before competition as

$$S_t = Q_{t^*}^{\mathrm{O}} p_{t^*}^{\mathrm{O}}.$$

In the following, let $\mathcal{T}_j = \{\mathcal{C}_{1,j}, \ldots, \mathcal{C}_{K_j,j}\}$ be the country specific pooled index set, where K_j represents the number of competition groups in country j. For each $l \in \{\mathrm{O,G,M}\}$ and $j \in \{\mathrm{US,Germany,Switzerland}\}$ we estimated the effect of the number of competitors on the percentage price change via the following linear regression

$$r_i^l = \alpha_j^l + \theta_{c(i)}^l + \mathbf{Z}_i^\top \boldsymbol{\beta}_j^l + \epsilon_i^l, \quad i \in \mathcal{T}_j, \quad c \in \{2, \ldots, C\},$$

where $\theta_{c(i)}^l$ represent the fixed effects for the number of competitors, with C being the maximum number of competitors, ϵ_i^l are mean zero noise terms, $\mathbf{Z}_i = (\mathrm{Time}_i, \mathrm{Quarters}_i, \mathrm{TherapeuticArea}_i, S_i)$ are vectors of control variables including, dummies for the date when the competition started, dummies for the quarters since the competition started, dummies for the therapeutical area and a continuous variable representing the sales before the competition started. Note: by construction $\theta_2^l = 0$.

The 95% confidence intervals for the number of competitor fixed effects were calculated in the classical way via

$$CI_{95\%}(\theta_c^l) = \hat{\theta}_c^l \pm 1.96\,\mathrm{SE}_{\text{CL-HC3}}(\hat{\theta}_c^l),$$

where $\mathrm{SE}_{\mathrm{CL\text{-}HC3}}\left(\hat{\theta}_c^l\right)$ were clustered and heteroskedasticity-consistent (HC3) standard errors. The standard errors were calculated via the vcovCL function from the sandwich R package with clusters on the active substance level and “HC3” as type argument.
